# Diagnostic and Prognostic Evaluation of Novel Biomarkers Compared to ESC 0/1 h and 0/3 h Algorithms in Patients with Suspected Non-ST-Elevation Myocardial Infarction

**DOI:** 10.3390/jcm14092957

**Published:** 2025-04-24

**Authors:** Mustafa Yildirim, Christian Salbach, Matthias Mueller-Hennessen, Norbert Frey, Evangelos Giannitsis

**Affiliations:** 1 Department of Internal Medicine III, Cardiology, University Hospital of Heidelberg, 69120 Heidelberg, Germany; 2 DZHK (German Centre for Cardiovascular Research), Standort Heidelberg/Mannheim, 69120 Heidelberg, Germany

**Keywords:** diagnosis, cardiac troponin, high sensitivity, cMyBP-C, acute coronary syndrome, emergency department

## Abstract

**(1) Background:** Prompt acute coronary syndrome (ACS) recognition remains challenging. This study evaluated the diagnostic and prognostic performance of novel biomarkers for non-ST-elevation myocardial infarction (NSTEMI). **(2) Methods:** Patients with suspected ACS presenting to Heidelberg University Hospital’s Emergency Department between August 2014 and February 2023 were analyzed. The biomarker panel included high-sensitivity cardiac troponin T (hs-cTnT), cardiac myosin-binding protein C (cMyBP-C), pro-B-type natriuretic peptide (proBNP), total N-terminal pro-B-type natriuretic peptide (t-NtproBNP), Angiotensin II (Ang2), Bone morphogenetic protein 10 (BMP10), Endothelial cell-specific molecule 1 (ESM1), fatty acid-binding protein 3 (FABP3), Fibroblast growth factor 23 (FGF23), Growth differentiation factor 15 (GDF15), and Copeptin. Negative predictive values (NPVs), sensitivities, and area under the curve (AUC) values were calculated for NSTEMI discrimination. Effectiveness and prognostic performance were assessed based on cardiovascular events at 30 days and 1 year. **(3) Results:** Of 1765 patients, 212 (12%) were diagnosed with NSTEMI. The European Society of Cardiology (ESC) 0/1 h and 0/3 h algorithms achieved sensitivities of 100% and 96.8%, NPVs of 100% and 99.3%, and effectiveness values of 54.8% and 66.0%. Hs-cTnT (AUC: 0.922) and cMyBP-C (AUC: 0.917) exhibited the highest diagnostic accuracy, followed by FABP3 (AUC: 0.759) and Copeptin (AUC: 0.624). Other biomarkers had lower performance (AUC: 0.516–0.617). At 1 year, event rates ranged from 0.0% to 3.4%, with the ESC algorithms demonstrating superior prognostic performance (0.8%, 2.4%). **(4) Conclusions:** The ESC 0/1 h and 0/3 h algorithms remain the most effective NSTEMI diagnostic strategies, balancing high sensitivity, prognostic reliability, and effectiveness. Among novel biomarkers, only cMyBP-C demonstrated comparable accuracy to hs-cTnT, supporting its potential as an adjunct to troponin assays.

## 1. Introduction

Acute coronary syndrome (ACS) poses a critical diagnostic challenge in emergency departments (EDs) worldwide, with chest pain as the predominant symptom, requiring rapid and accurate evaluation to improve outcomes [1,2,3]. International guidelines endorse high-sensitivity cardiac troponins (hs-cTn) as a cornerstone of ACS care, streamlining triage and reducing diagnostic uncertainty [2,3,4].

However, hs-cTn assays have limitations, including delayed elevation in the first 3 h after myocardial ischemia onset, resulting in reduced sensitivity in early presenters (<3 h), and challenges in patients with chronic renal or heart failure due to elevated baseline levels [2,5,6,7,8,9,10]. As a result, European Society of Cardiology (ESC) guidelines advise caution in using hs-cTn-based pathways for early presenters, emphasizing the need for additional biomarkers to enhance early diagnosis and improve care in this critical window [2]. Efforts to develop novel, highly accurate biomarkers aim to overcome these limitations, facilitating earlier diagnosis, reducing mortality, and mitigating complications, either as standalone tools or in combination with standard cardiac troponins (cTn) and hs-cTn assays [11,12,13,14,15,16,17].

Given these limitations, there is a critical need to evaluate emerging biomarkers for their potential to improve early non-ST-elevation myocardial infarction (NSTEMI) detection, rapid rule-out strategies, and risk stratification. This study directly compares the diagnostic and prognostic accuracy of established ESC 0/1 h and ESC 0/3 h algorithms with several novel biomarkers—including high-sensitivity cardiac troponin T (hs-cTnT), cardiac myosin-binding protein C (cMyBP-C), pro-B-type natriuretic peptide (proBNP), N-terminal pro-B-type natriuretic peptide (NtproBNP), Copeptin, and others—for timely diagnosis, the rapid rule-out of NSTEMI, and the prediction of outcomes at 12 months.

## 2. Materials and Methods

### 2.1. Study Population

The study population consisted of patients presenting with acute symptoms suggestive of ACS to the ED of Heidelberg University Hospital from August 2014 to February 2023. This study represents a retrospective observational analysis. While patient enrollment and sample collection were conducted prospectively within a registry, subsequent biomarker measurements were performed retrospectively from frozen samples stored at −80 °C until analysis. The comprehensive biomarker panel included high-sensitivity cardiac troponin T (hs-cTnT), cMyBP-C, proBNP, total N-terminal pro-B-type natriuretic peptide (t-NtproBNP), Angiotensin II (Ang2), Bone morphogenetic protein 10 (BMP10), Endothelial cell-specific molecule 1 (ESM1), fatty acid-binding protein 3 (FABP3), Fibroblast growth factor 23 (FGF23), Growth differentiation factor 15 (GDF15), NtproBNP, and Copeptin. Diagnosis encompassed NSTEMI, unstable angina pectoris (UAP), along with non-coronary cardiac and non-cardiac conditions (non-ACS).

Patients were eligible for enrollment in the prospective all-comer registry if they presented with clinically suspected ACS, defined by a broad spectrum of symptoms including typical or atypical chest pain, dyspnea, or other ischemic equivalents. Exclusion criteria at the registry level included (1) primary pulmonary disease without suspected ACS; (2) traumatic chest pain due to preceding thoracic injury; (3) chronic hemodialysis; (4) inadequate command of German or English language; and (5) permanent residence outside Germany.

The present retrospective study was restricted to a well-characterized cohort of patients with complete biomarker data. For this purpose, patients with ST-elevation myocardial infarction (STEMI) and those lacking baseline (0 h) measurements of the extended biomarker panel (cMyBP-C, proBNP, t-NtproBNP, Ang2, BMP10, ESM1, FABP3, FGF23, GDF15, Copeptin) were excluded.

NSTEMI diagnosis followed the Fourth Universal Definition criteria (1), requiring elevated hs-cTnT levels above the 99th percentile, coupled with a rising or falling pattern and clinical evidence of myocardial ischemia. Final diagnoses were confirmed by an expert committee comprising two independent cardiologists (MMH, MY), with consultation from a third cardiologist (EG) in cases of disagreement. Diagnostic and therapeutic decisions were made at the discretion of the attending physician. The study was carried out according to the principles of the Declaration of Helsinki and approved by the local ethics committee of the medical faculty of Heidelberg University (approval code: S-351/2015; approval date: 7 September 2015). Written informed consent was obtained from all patients. As this study was part of an all-comer real-world registry, only patients who provided informed consent were included. Patients who declined consent were not enrolled in the study, and no additional blood samples were obtained from this group. The trial was registered on ClinicalTrials.gov (Clinical Trails.gov Identifier: NCT06128317).

### 2.2. Laboratory Analysis

Blood samples were collected upon patient arrival in the ED using EDTA-plasma tubes. After centrifugation, plasma samples were immediately analyzed for hs-cTnT, NT-proBNP, and Copeptin, while aliquots for additional biomarker analyses (cMyBP-C, proBNP, t-NtproBNP, Ang2, BMP10, ESM1, FABP3, FGF23, and GDF15) were stored at −80 °C and later assayed in a blinded manner in a laboratory at Roche Diagnostics (Penzberg, Germany). The measurement of hs-cTnT in plasma samples was performed using the Elecsys^®^ Troponin T high-sensitivity assay (Roche Diagnostics) on a cobas e411 immunoassay analyzer, with limit of blank (LoB), limit of detection (LoD), 10% coefficient of variation (CV), and 99th percentile cut-off values at 3 ng/L, 5 ng/L, 13 ng/L and 14 ng/L, respectively [18,19]. The ESC 0/3 h algorithm was systematically applied to all patients enrolled prior to the publication of the 2015 ESC Guidelines for NSTE-ACS, after which the ESC 0/1 h algorithm was systematically implemented [20]. A third hs-cTnT measurement was only obtained in patients who were triaged into the observational zone according to the applicable ESC guidelines per ESC 0/1 h algorithm [21]. No sex-specific hs-cTnT cut-off values were applied. NT-proBNP (Siemens Atellica^®^ IM NT-proBNP; Siemens Healthineers, Erlangen, Germany) was measured along with the performance of routine blood chemistry using fresh plasma. The Copeptin in plasma samples at baseline (0 h) was measured with the Copeptin proAVP assay on the KRYPTOR compact plus (BRAHMS Thermo Fisher Scientific, Hennigsdorf, Germany), with a detection limit, a precision of 20% CV, and a 95th percentile cut-off of 0.69 pmol/L, 1.08 pmol/for and 9.8 pmol/L, respectively [22,23].

### 2.3. Outcome Measures

The primary outcome was the diagnostic performance of biomarkers for detecting NSTEMI and their effectiveness in rapidly ruling out NSTEMI. The secondary outcome was a composite endpoint of all-cause death, myocardial infarction (MI), and stroke at 30 days and 1 year. The prognostic performance of each biomarker-based rule-out strategy was assessed by evaluating their ability to exclude AMI and stratify risk for this composite endpoint. GRACE risk scores were calculated according to established methods [24,25]. Follow-up was accomplished using telephone, questionnaire, patient’s hospital notes, the family physician’s records, and the municipal registry on vital status.

### 2.4. Statistical Methods

Continuous variables are presented as median with interquartile range (IQR) for a non-normal distribution or as means ±95% confidence intervals (CI) for normally distributed data. For comparison of continuous parameters, the Mann–Whitney U-test was used, whereas a Chi-square test was applied for categorical parameters.

Discrimination power was assessed using the area under the receive–operating characteristics curve (AUC) for each biomarker using the DeLong test [26], employing 1000 stratified bootstrap replicates to calculate CIs. All biomarker analyses, including ROC curve generation, were consistently based on baseline (0 h) biomarker measurements. To identify thresholds for effective rule-in and rule-out strategies, optimal ROC-derived cut-off values were determined for each biomarker based on predefined targets of sensitivity ≥99.5% and specificity >95%. Diagnostic metrics, including sensitivity, specificity, positive predictive value (PPV), and negative predictive value (NPV), were calculated for each biomarker and strategy.

Adjusted analyses were performed using multivariable logistic regression models to account for age, sex, the history of myocardial infarction, diabetes, hypertension, hypercholesterolemia, smoking status (past or current), and GRACE score. Prognostic performance, regarding mortality and the composite endpoint (all-cause death, MI, stroke) at 30 days and 1 year, was evaluated using Cox regression models and Kaplan–Meier survival curves. To assess the robustness of the prognostic findings, a sensitivity analysis limited to patients with confirmed NSTEMI was performed, using univariable Cox regression models for each biomarker, with a 1-year follow-up as the dependent variable. All hypothesis tests were two-tailed, with *p*-values < 0.05 considered statistically significant. Statistical analyses were performed using R software (version 4.3.0; R Foundation for Statistical Computing, Vienna, Austria).

## 3. Results

### 3.1. Baseline Characteristics

**Figure 1:**
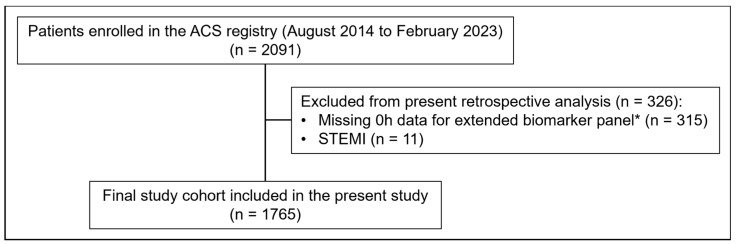
Flow diagram of included and excluded patients. Flowchart illustrates selection process for final study cohort from ACS registry between August 2014 and February 2023. Patients were excluded from the present retrospective analysis if they had ST-elevation myocardial infarction (STEMI) or lacked 0 h measurements from the extended biomarker panel. Abbreviations: ACS, acute coronary syndrome; STEMI, ST-elevation myocardial infarction. * extended biomarker panel: cMyBP-C, proBNP, t-NtproBNP, Ang2, BMP10, ESM1, FABP3, FGF23, GDF15, Copeptin.

From an initial pool of 2091 patients presenting to the ED with suspected ACS, 1765 were included in the final analysis after excluding cases with incomplete baseline biomarker data or ST-elevation myocardial infarction (STEMI). Patient inclusion required written informed consent and timely sample collection. In a busy tertiary care ED with frequent overcrowding, this requirement naturally limited recruitment despite a large overall patient volume. Figure 1 outlines the selection process and details the final study cohort. Among these, 212 (12%) were diagnosed with NSTEMI, while the remaining 1553 (88%) patients received other diagnoses, including 515 (29.2%) with unstable angina pectoris (UAP) and 1038 (58.8%) with non-ACS conditions. The ESC 0/1 h algorithm was applied in 66.7% of patients (1178 of 1765), and the ESC 0/3 h algorithm was applied in 74.1% of patients (1308 of 1765) within the total study cohort. Follow-up was completed at both 30 days and 1 year (100%) for all included patients. NSTEMI patients exhibited distinct clinical and demographic characteristics compared to those with other diagnoses, as shown in Table 1. They tended to be older (median age 68 vs. 66 years), predominantly male (77.8% vs. 59.8%), and were more likely to present with chest pain (95.8% vs. 79.3%) and higher GRACE risk scores (median 111 vs. 94, *p* < 0.0001). Moreover, NSTEMI patients had higher prevalences of comorbidities, including previous myocardial infarction (27.8% vs. 18.0%), diabetes mellitus (26% vs. 16%), and arterial hypertension (78% vs. 72%), as well as higher rates of current smoking (25% vs. 17%). Regarding clinical presentation, NSTEMI patients had significantly elevated biomarker levels at baseline compared to those with other diagnoses, including median concentrations of cMyBP-C (238.0 vs. 11.4 ng/L, *p* < 0.0001), hs-cTnT (62 vs. 9 ng/L, *p* < 0.0001), NT-proBNP (864 vs. 181 ng/L, *p* < 0.0001), and Copeptin (8.8 vs. 5.7 pmoL/L, *p* < 0.0001). Additional details on baseline biomarker distributions are summarized in Supplementary Table S1.

### 3.2. Discriminatory Ability of Biomarkers

**Figure 2:**
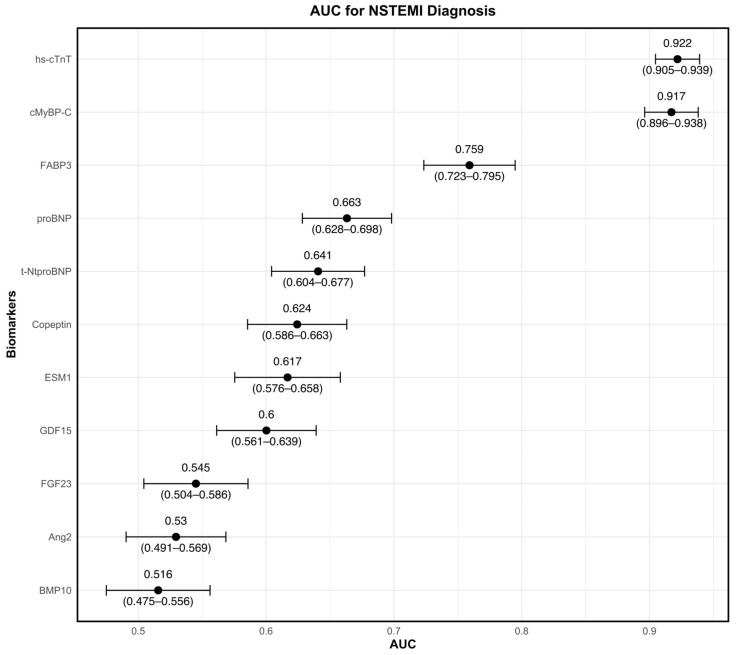
Diagnostic performance of biomarkers for NSTEMI: AUC analysis. Abbreviations: Ang2, Angiotensin II; AUC, area under the curve; BMP10, Bone morphogenetic protein 10; cMyBP-C, cardiac myosin-binding protein C; ESM1, Endothelial cell-specific molecule 1; FABP3, fatty acid-binding protein 3; FGF23, Fibroblast growth factor 23; GDF15, Growth differentiation factor 15; Hs-cTnT, high-sensitivity cardiac troponin T; NSTEMI, non-ST-myocardial infarction; proBNP, pro-B-type natriuretic peptide; t-NtproBNP, total N-terminal pro-B-type natriuretic peptide.

**Figure 3:**
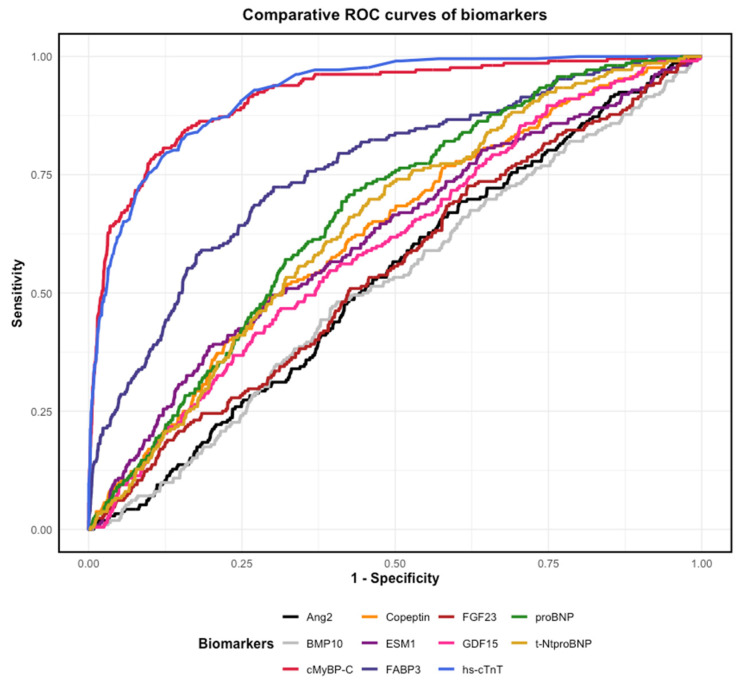
Comparative ROC curves of biomarkers for NSTEMI diagnosis. Abbreviations: Ang2, Angiotensin II; BMP10, Bone morphogenetic protein 10; cMyBP-C, cardiac myosin-binding protein C; ESM1, Endothelial cell-specific molecule 1; FABP3, fatty acid-binding protein 3; FGF23, Fibroblast growth factor 23; GDF15, Growth differentiation factor 15; Hs-cTnT, high-sensitivity cardiac troponin T; NSTEMI, non-ST-myocardial infarction; proBNP, pro-B-type natriuretic peptide; ROC, receiver operating characteristic; t-NtproBNP, total N-terminal pro-B-type natriuretic peptide.

**Figure 4:**
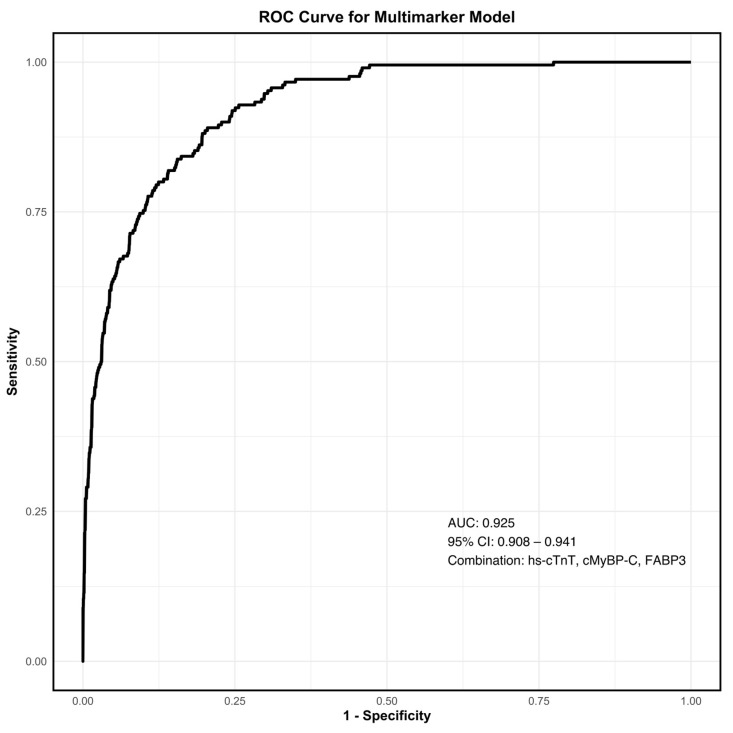
ROC curve showing diagnostic performance of multimarker model incorporating cMyBP-C, hs-cTnT, and FABP3 for diagnosis of NSTEMI. Abbreviations: cMyBP-C, cardiac myosin-binding protein C; FABP3, fatty acid-binding protein 3; Hs-cTnT, high-sensitivity cardiac troponin T; NSTEMI, non-ST-myocardial infarction; ROC, receiver operating characteristic.

The diagnostic performance of each biomarker for NSTEMI, as reflected by AUC, is summarized in Table 2. Hs-cTnT demonstrated the highest discriminatory ability, with an AUC of 0.922 (95% CI, 0.905–0.939), closely followed by cMyBP-C, with an AUC of 0.917 (95% CI, 0.896–0.938; *p* = 0.5849 for comparison with hs-cTnT). FABP3 achieved the third highest AUC at 0.759 (95% CI, 0.723–0.795), while proBNP, t-NtproBNP, and Copeptin exhibited moderate discriminatory power with AUCs of 0.663, 0.641, and 0.624, respectively. Biomarkers with lower diagnostic performance included Ang2, BMP10, FGF23, GDF15 and ESM1, all with AUCs ≤ 0.6. Figure 2 and Figure 3 illustrate the diagnostic utility of the biomarkers, with Figure 2 displaying individual AUCs and Figure 3 providing comparative ROC curves for NSTEMI diagnosis. To evaluate the incremental diagnostic benefit of combining the three biomarkers with the highest individual AUCs, we assessed a multimarker model incorporating cMyBP-C, hs-cTnT, and FABP3 (Figure 4). The multimarker approach demonstrated an AUC of 0.925 (95% CI, 0.908–0.941), compared to hs-cTnT alone, with an AUC of 0.922 (95% CI, 0.905–0.939).

### 3.3. Adjusted Analyses Accounting for Confounders

After adjusting for age, gender, cardiovascular risk factors, and GRACE score, hs-cTnT retained the highest discriminatory ability, with an adjusted AUC of 0.898 (95% CI, 0.875–0.921), followed closely by cMyBP-C with an AUC of 0.874 (95% CI, 0.847–0.900). FABP3 also demonstrated strong diagnostic performance after adjustment (AUC: 0.760, 95% CI: 0.728–0.793). In contrast, other biomarkers, including proBNP, t-NtproBNP, and Ang2, showed limited diagnostic value after adjustment, with AUCs ranging from 0.708 to 0.724. These findings are summarized in Table 3.

### 3.4. Biomarker Cut-Off Values and Diagnostic Utility

Table 3 summarizes the ROC-derived optimal cut-off values for effective rule-in and rule-out strategies, along with their respective diagnostic performance. Hs-cTnT demonstrated the highest diagnostic utility, with a rule-in threshold of >42.0 ng/L (PPV 63.6%) and a rule-out threshold of ≤7.9 ng/L (NPV 99.9%). Similarly, cMyBP-C exhibited a rule-in threshold of >117.8 ng/L (PPV 64.2%) and a rule-out threshold of ≤2.3 ng/L (NPV 99.3%). FABP3 also showed strong performance, with rule-in and rule-out thresholds of >61.40 ng/mL (PPV 90.6%) and ≤12.79 ng/mL (NPV 97.6%), respectively. In contrast, proBNP and t-NtproBNP demonstrated modest rule-in utility, with thresholds of >3801.3 pg/L (PPV 19.8%) and >9942.3 ng/L (PPV 16.0%), but more robust rule-out values of ≤9.1 pg/L (NPV 98.1%) and ≤86.7 ng/L (NPV 97.8%). Among the remaining biomarkers, Copeptin, ESM1, and GDF15 exhibited moderate utility, with NPVs ranging from 75% to 96%, while Ang2, BMP10, and FGF23 showed limited diagnostic relevance (AUC ≤ 0.6), reflecting insufficient sensitivity or specificity in distinguishing NSTEMI from other conditions.

### 3.5. Diagnostic Performance of Single Biomarker Strategies Compared to Serial hs-cTnT as the Reference (ESC 0/1 h and ESC 0/3 h Algorithms)

The diagnostic performance and clinical utility (defined as the proportion of patients correctly ruled in or ruled out, termed “effectiveness”) of single-biomarker strategies were evaluated using serial hs-cTnT measurements, with ESC recommendations as the reference.

Single-baseline hs-cTnT at the 99th percentile diagnostic cut-off (≤14 ng/L) achieved high sensitivity and NPVs (both 92.5%) with substantial effectiveness (64.6%). The ESC 0/1 h algorithm provided the highest sensitivity (100%) and NPV (100%) but had comparatively lower effectiveness (54.8%). The ESC 0/3 h algorithm yielded slightly lower sensitivity (96.8%) and NPV (99.3%) but achieved the highest effectiveness (66.0%) (Table 4).

Among novel biomarkers, cMyBP-C showed the second-highest diagnostic accuracy after hs-cTnT but limited effectiveness (26.9%). FABP3, despite good diagnostic performance (third-highest AUC), exhibited low effectiveness (2.0%). The remaining novel biomarkers (proBNP, t-NtproBNP, Ang2, BMP10, ESM1, FGF23, and GDF15) showed even lower effectiveness, ranging between 0% and 13.3% (Table 2).

Overall, compared to novel single-biomarker approaches, the established ESC serial hs-cTnT algorithms (0/1 h and 0/3 h) provided superior clinical utility, balancing high sensitivity and NPVs with considerably higher effectiveness.

### 3.6. Prognostic Performance of Single-Biomarker Strategies Compared to Serial hs-cTnT Algorithms (ESC 0/1 h and ESC 0/3 h Algorithms)

**Figure 5:**
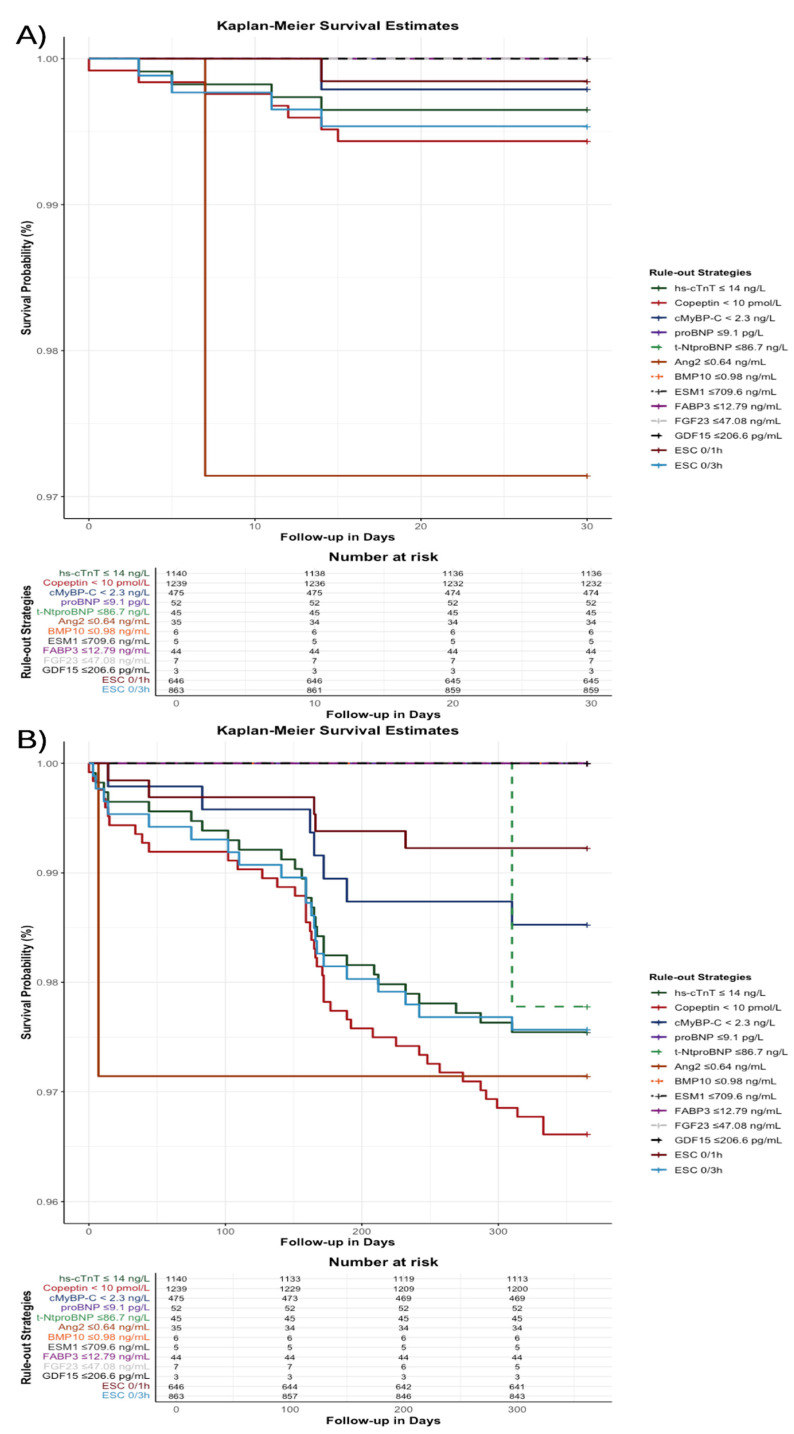
Kaplan–Meier survival estimates for biomarker-based rule-out strategies at 30 days (**A**) and 1 year (**B**) compared to the ESC 0/1 h and ESC 0/3 h algorithms. Abbreviations: Angiotensin II; BMP10, Bone morphogenetic protein 10; cMyBP-C, cardiac myosin-binding protein C; ESM1, Endothelial cell-specific molecule 1; FABP3, fatty acid-binding protein 3; FGF23, Fibroblast growth factor 23; GDF15, Growth differentiation factor 15; Hs-cTnT, high-sensitivity cardiac troponin T; proBNP, pro-B-type natriuretic peptide; ROC, receiver operating characteristic; t-NtproBNP, total N-terminal pro-B-type natriuretic peptide.

Table 5 summarizes the prognostic performance of each biomarker-based rule-out strategy for predicting the composite endpoint (all-cause death, MI, or stroke) at 30 days and 1 year. The 30-day event rates for single-biomarker strategies were generally low (ranging between 0.0% and 2.9%), with Ang2 having the highest event rate (2.9%), compared to 0.2% and 0.5% for ESC 0/1 h and ESC 0/3 h algorithms, respectively. At 1 year, event rates for single biomarkers ranged from 0.0% to 3.4%, whereas event rates for ESC 0/1 h and 0/3 h algorithms were 0.8% and 2.4%, respectively (Table 5). Overall, the established ESC serial hs-cTnT algorithms (0/1 h and 0/3 h) provided superior prognostic performance compared to single-biomarker strategies. Figure 5A presents the 30-day Kaplan–Meier survival curves and Figure 5B presents the 1-year Kaplan–Meier survival curves for single-marker strategies compared to the ESC 0/1 h and ESC 0/3 h algorithms. To confirm the robustness of these findings, sensitivity analysis limited to patients with confirmed NSTEMI was performed. In this subgroup, higher baseline concentrations of hs-cTnT, cMyBP-C, FABP3, GDF15, and Copeptin remained significantly associated with adverse 1-year outcomes (Supplementary Table S2)

## 4. Discussion

In this study, we directly compared the diagnostic and prognostic performance of several novel biomarkers against the established ESC 0/1 h and ESC 0/3 h algorithms in a large, well-characterized cohort of patients presenting with suspected acute coronary syndrome.

We demonstrate five important findings:

Firstly, the ESC 0/1 h and 0/3 h algorithms demonstrated the best diagnostic performance, achieving high sensitivity (100% and 96.8%, respectively) and negative predictive value (100% and 99.3%) while maintaining high effectiveness (54.8% and 66.0%). Among novel biomarkers, hs-cTnT and cMyBP-C demonstrated the highest discriminative abilities for diagnosing NSTEMI within the biomarker panel, with AUCs of 0.922 and 0.917, respectively. These findings align with a substudy of the APACE trial (Advantageous Predictors of Acute Coronary Syndrome Evaluation), which reported comparable discriminatory performance for cMyBP-C (AUC: 0.924) and hs-cTnT (AUC: 0.927) in detecting myocardial infarction, surpassing conventional troponins such as s-cTnI (AUC: 0.909) [27]. In contrast, Kaier et al. [28] reported superior discriminatory power of cMyBP-C (AUC: 0.839) over hs-cTnT (AUC: 0.813), likely due to variations in study design, including differences in patient selection criteria, the timing of blood sample collection, or adjudication processes for NSTEMI diagnosis. Additionally, differences in the demographic and clinical characteristics of the study populations or the analytical platforms used to measure cMyBP-C and hs-cTnT could have influenced the observed results. The diagnostic utility of hs-cTnT for NSTEMI diagnosis is well-established in the literature, and its exceptional performance in our study reaffirms its central role in clinical practice [2]. Additionally, beyond the initial diagnostic assessment, hs-cTn measurements post-percutaneous coronary intervention (PCI) provide significant prognostic insights in NSTEMI patients. Recent evidence highlights that periprocedural myocardial injury and type 4a myocardial infarction, identified through substantial post-PCI troponin elevations, occur frequently and correlate with markedly worse clinical outcomes, including increased rates of mortality and cardiovascular events at one year [29]. Therefore, routine hs-cTn evaluation following PCI may enhance risk stratification and improve clinical decision-making by identifying patients at elevated risk for adverse events. However, our aim was not to challenge the established role of hs-cTnT but to explore the potential of additional biomarkers, such as cMyBP-C, to complement hs-cTnT in refining diagnostic strategies. This approach is particularly relevant in clinical scenarios where hs-cTnT has limitations, such as early presenters or patients with chronically elevated troponin levels due to underlying conditions [27,30,31,32]. While repeat troponin measurements are often required in cases of high clinical suspicion or early presentation, our findings suggest that cMyBP-C could serve as a valuable adjunct biomarker. With its comparable discriminatory ability to hs-cTnT, cMyBP-C may refine decision-making and improve early triage in situations where time 0 hs-cTnT alone is insufficient. While our study does not propose replacing current algorithms, it lays the groundwork for future research on incorporating cMyBP-C into established pathways to optimize diagnostic workflows. To ensure the robustness of these findings, we performed adjusted analyses accounting for traditional cardiovascular risk factors and other confounders, including age, sex, history of myocardial infarction, diabetes, hypertension, hypercholesterolemia, smoking status, and the GRACE score. Even after adjustment, hs-cTnT retained the highest discriminatory ability with an AUC of 0.898 (95% CI: 0.875–0.921), followed closely by cMyBP-C with an AUC of 0.874 (95% CI: 0.847–0.900). Notably, FABP3 also maintained strong performance after adjustment (AUC: 0.760, 95% CI: 0.728–0.793). These findings confirm that the diagnostic performance of these biomarkers is independent of traditional risk factors and highlights their potential utility as reliable tools for NSTEMI diagnosis. Supporting this, a prior investigation [33] involving 1495 patients found that the cMyBP-C 0/1 h algorithm provided diagnostic performance comparable to the ESC-recommended hs-cTnT/I 0/1 h algorithms. Interestingly, cMyBP-C demonstrated superior rule-out capabilities using a single 0 h sample, though its overall performance aligned closely with hs-cTnT and hs-cTnI after completion of the 0/1 h-algorithm. While cMyBP-C offers promising potential as an adjunct biomarker to complement hs-cTnT, its primary value lies in enhancing triage efficiency, reducing diagnostic uncertainty, and streamlining emergency department workflows. Although these improvements may streamline system-level outcomes, such as healthcare resource utilization, their direct impact on individual patient outcomes—such as reducing mortality or major complications—may be limited, given the established pathways for urgent management of high-risk NSTEMI patients. While cMyBP-C is predominantly cardiac-specific, its specificity can be influenced by conditions like chronic kidney disease or a history of heart failure, where elevated baseline levels have been reported in previous studies due to ongoing cardiomyocyte stress or reduced clearance. This aligns with findings from Kaier et al. and the APACE substudy [27], which noted higher cMyBP-C concentrations in these populations. Although these comorbidities are underrepresented in our cohort, our study provides important data on the utility of cMyBP-C in a broader ACS population without such confounders. Unlike previous studies comparing cMyBP-C and hs-cTnT alone [27], our study comprehensively evaluates hs-cTnT, cMyBP-C, proBNP, t-NtproBNP, Ang2, BMP10, ESM1, FABP3, FGF23, GDF15, and Copeptin within the same cohort, offering unique insights into their comparative diagnostic value under consistent clinical and methodological conditions.

Additionally, building on these findings, we evaluated the incremental diagnostic benefit of a multimarker model combining cMyBP-C, hs-cTnT, and FABP3. This model demonstrated an AUC of 0.925 (95% CI, 0.908–0.941), compared to hs-cTnT alone, which had an AUC of 0.922 (95% CI, 0.905–0.939). Although the absolute gain was limited, these findings suggest the potential clinical utility of cMyBP-C as an adjunct biomarker, particularly in challenging clinical scenarios such as early presenters or borderline hs-cTnT elevations, where established algorithms may be insufficient [27,30,31,32]. While not replacing hs-cTnT, cMyBP-C demonstrates potential as an adjunct biomarker, particularly in cases where current algorithms may be less reliable. Future research should focus on validating its real-time clinical utility, with emphasis on developing point-of-care assays to facilitate rapid decision-making in emergency settings. Additionally, integrating cMyBP-C into accelerated diagnostic protocols (e.g., ESC 0/1 h algorithms) alongside hs-cTnT and clinical risk scores such as GRACE may refine rule-in and rule-out strategies, particularly in challenging cases like early presenters. Prospective multicenter studies are warranted to confirm its incremental diagnostic value, assess patient-centered outcomes, and evaluate effects on clinical workflows and resource utilization.

Secondly, our findings regarding FABP3 align with the FAME-ER (Fatty Acid Binding Protein in Myocardial Infarction Evaluation in the Emergency Room) study, which reported an AUC of 0.73 for heart-type fatty acid-binding protein (H-FABP) ELISA in a cohort of 543 patients [34]. These results reinforce previous evidence that FABP3 has limited diagnostic utility in ACS, lacking sufficient reliability both as a standalone marker and when combined with hs-cTn for diagnosing AMI [34,35]. This limitation may stem from FABP3’s relatively low specificity, as it is expressed in both cardiac and skeletal muscle, and its rapid renal clearance, resulting in a short plasma half-life [36,37]. These factors reduce its effectiveness, particularly in late presenters, thereby limiting its clinical applicability.

Thirdly, in our study, Copeptin demonstrated limited discriminatory ability as a stand-alone biomarker for diagnosing NSTEMI, with an AUC of 0.624, reflecting its lack of clinical specificity. However, when combined with cardiac troponins (cTn), Copeptin has shown added value in several studies, supporting its use as an adjunctive marker to facilitate early and safe discharge decisions in low- to intermediate-risk patients [13,14,22,38,39]. Our findings align with these observations, underscoring that Copeptin’s primary utility lies in its role as a complementary biomarker within multimarker diagnostic strategies, rather than as a stand-alone tool. Therefore, Copeptin has received a recommendation for the rule-out of an NSTEMI when used together with cTn [20].

Fourthly, while the optimal ROC-derived cut-off value for cMyBP-C for rule-out in our cohort differed from that reported in the APACE substudy [27], the rule-in thresholds were comparable (>117.8 ng/L in our cohort vs. 120 ng/L in the APACE substudy). In the APACE substudy, cMyBP-C achieved a sensitivity of 99.6% and a negative predictive value (NPV) of 99.8% at a rule-out threshold of 10 ng/L. In contrast, our cohort demonstrated equivalent sensitivity (99.6%) with a slightly lower NPV (99.3%) at a more stringent rule-out cut-off of ≤2.3 ng/L. These variations likely reflect differences in study populations, assay platforms, or analytical methodologies, such as calibration standards or detection limits, potentially influencing the observed results.

Finally, beyond diagnostic accuracy, our study highlights the strong prognostic performance and clinical effectiveness of the ESC 0/1 h and 0/3 h algorithms. These strategies achieved low event rates at 30 days (0.2% and 0.5%) and 1 year (0.8% and 2.4%), reinforcing their reliability in both short- and long-term risk assessment. In contrast, single-biomarker strategies showed variable and often limited prognostic utility, with 30-day event rates ranging from 0.0% to 0.6%, except for Ang2 (2.9%), and 1-year rates ranging from 0.0% to 3.4%. No biomarker clearly outperformed the ESC algorithms in prognostic accuracy.

These findings confirm that the ESC algorithms provide an optimal balance between diagnostic accuracy, prognostic reliability, and clinical applicability, supporting their role as the standard of care. While some novel biomarkers may hold promise, particularly for specific subgroups, their added value beyond established hs-cTnT-based strategies remains uncertain. Future research should focus on whether integrating additional biomarkers—such as cMyBP-C—alongside hs-cTnT and clinical risk scores (e.g., GRACE) could further refine risk stratification and optimize patient triage. However, until robust evidence demonstrates clear prognostic benefits, the ESC 0/1 h and 0/3 h algorithms remain the most effective and validated strategies for both early and long-term risk assessment in patients with suspected ACS.

### 4.1. Limitations

While our study offers valuable insights into diagnostic and prognostic biomarker performance, it is crucial to address several limitations. Firstly, its single-center nature in Germany may limit generalizability to other regions or healthcare systems. Secondly, the exclusion of patients with terminal kidney failure on renal replacement therapy restricts insights into biomarker performance in this subgroup. While cMyBP-C demonstrated high discriminatory power, its specificity may be affected by non-ischemic conditions like heart failure, chronic kidney disease, or sepsis, which can elevate baseline levels [27,33,40]. Additionally, analytical challenges, such as protein degradation or assay variability, could influence measurements. These factors underscore the need for assay standardization and further validation in diverse populations to confirm its robustness as a diagnostic tool. Thirdly, while blood samples for biomarkers were collected upon admission and processed consistently, they were analyzed in batches at an external laboratory, which does not reflect real-time clinical practice. This limitation impacts the immediate clinical utility of certain biomarkers, particularly those, such as cMyBP-C, requiring rapid turnaround times for acute diagnosis. Future studies should focus on validating these biomarkers in real-time settings and developing point-of-care assays to optimize their diagnostic applicability in acute care workflows. Fourthly, biomarker performance at predefined ROC-optimized cut-off values, selected a priori to maximize sensitivity and NPVs, may introduce bias and thus overestimate real-world clinical effectiveness. Fifthly, potential bias against cMyBP-C and other biomarkers due to clinical adjudication based on hs-cTnT warrants consideration. Finally, while hs-cTnT is widely available, the research platform used for measuring other biomarkers may hinder routine clinical use.

### 4.2. Strengths

Despite these limitations, our study exhibits notable strengths that fortify the credibility and applicability of its findings. Firstly, its rigorous design and adherence to standardized protocols ensure the accuracy and reliability of the results. Secondly, being conducted within a real-world clinical setting, the study’s findings directly apply to routine practice in EDs, enhancing its external validity. Thirdly, the incorporation of a sizable patient cohort bolsters the statistical power of the study, enabling more robust conclusions. These combined strengths significantly enhance the significance and relevance of our study in guiding clinical decision-making regarding ACS management.

## 5. Conclusions

In conclusion, the ESC 0/1 h and 0/3 h algorithms demonstrated the best overall performance, achieving an optimal balance between diagnostic accuracy, prognostic reliability, and clinical effectiveness. These established strategies provided high sensitivity and negative predictive value while maintaining low short- and long-term event rates, reinforcing their role as the gold standard for NSTEMI diagnosis.

Among the novel biomarkers assessed, only cMyBP-C showed promise, demonstrating comparable discriminatory power to hs-cTnT. While hs-cTnT remains the cornerstone of NSTEMI diagnosis, cMyBP-C may serve as a valuable adjunct, particularly in challenging clinical scenarios such as early presenters or patients with chronically elevated troponin levels. However, further independent validation is needed before its integration into routine clinical practice.

Future research should focus on evaluating cMyBP-C within multimarker strategies, particularly in combination with hs-cTnT, to determine whether it can enhance risk stratification and optimize emergency department triage. Additionally, prospective studies are warranted to assess the real-world impact of multimarker approaches on clinical decision-making and patient outcomes.

## Figures and Tables

**Table 1:** Baseline characteristics of all patients and stratified by diagnosis: NSTEMI vs. other diagnoses.

	NSTEMI (n = 212)	Other Diagnoses (n = 1553)	*p* Value *
Age [y], median (IQR)	68 (59–78)	66 (54–75)	0.0001
Female gender, n (%all)	47 (22.2)	624 (40.2)	<0.0001
Heart rate [bpm], median (IQR)	77 (66–87)	77 (66–89)	0.8524
Systolic pressure [mmHg], median (IQR)	153 (141–170)	150 (137–164)	0.2900
GRACE score, median (IQR)	111 (93–130)	94 (71–117)	<0.0001
Symptoms			
Time since onset < 3 h, n (%all)	20 (9.4)	216 (13.9)	<0.0001
Chest pain, n (%all)	203 (95.8)	1231 (79.3)	<0.0001
Dyspnea, n (%all)	102 (48.1)	642 (41.3)	0.2955
History			
Myocardial infarction, n (%all)	59 (27.8)	282 (18)	0.0008
Congestive heart failure, n (%all)	10 (3)	125 (8)	0.0898
Smoking current, n (%all)	53 (25)	261 (17)	<0.0001
Smoking past, n (%all)	62 (29)	575 (37)	0.0163
Art. hypertension, n (%all)	165 (78)	1114 (72)	0.0623
Diabetes mellitus, n (%all)	54 (26)	244 (16)	0.0017
Dyslipidemia, n (%all)	130 (61)	811 (52)	0.0128
Renal disease, n (%all)	34 (16)	192 (12)	0.1683
COPD, n (%all)	19 (9)	106 (7)	0.6271
Atrial fibrillation, n (%all)	22 (10)	334 (22)	0.0006
Coronary Angiography and Revascularization			
Coronary angiography, n (%all)	202 (95.3)	403 (26.0)	<0.0001
Coronary stenosis ≥70%, n (%all)	186 (87.7)	241 (15.5)	<0.0001
Revascularisation, n (%all)	168 (79)	155 (10)	<0.0001
PCI/stent, n (%all)	146 (68.9)	120 (7.7)	<0.0001
CABG, n (%all)	22 (10)	35 (2)	<0.0001
Laboratory			
cMyBP-C [ng/L], median (IQR)	238.0 (72.7–1019.0)	11.4 (5.3–26.6)	<0.0001
hs-cTnT 0 h [ng/L], median (IQR)	62 (28–184)	9 (5–15)	<0.0001
Creatinine [mg/L], median (IQR)	0.9 (0.8–1.1)	0.9 (0.7–1.0)	0.0015
NT-proBNP [ng/L], median (IQR)	864 (320–2159)	181 (66–989)	<0.0001
Total NT-proBNP [ng/L], median (IQR)	1948.9 (773.4–4411.8)	873.1 (329.8–2629.6)	<0.0001
proBNP [pg/L], median (IQR)	568.7 (170.0–1548.5)	950.5 (53.6–768.0)	<0.0001
Ang2 [ng/mL], median (IQR)	1.72 (1.24–2.48)	1.56 (1.15–2.37)	0.1379
BMP10 [ng/mL], median (IQR)	1.90 (1.52–2.16)	1.76 (1.48–2.17)	0.4509
ESM1 [ng/mL], median (IQR)	2372.8 (1652.8–4127.3)	1843.8 (1429.8–2729.1)	<0.0001
FABP3 [ng/mL], median (IQR)	41.0 (30.5–64.3)	26.3 (20.6–35.4)	<0.0001
FGF23 [ng/mL], median (IQR)	117.0 (94.3–167.9)	127.7 (97.2–200.4)	0.0299
GDF15 [pg/mL], median (IQR)	1339.7 (869.3–2363.0)	1074.3 (669.1–1727.1)	<0.0001
Copeptin [pmoL/L], median (IQR)	8.8 (4.9–16.7)	5.7 (3.5–11.1)	<0.0001

Abbreviations: Ang2, Angiotensin II; BMP10, Bone morphogenetic protein 10; bpm, beats per minute; CABG, coronary artery bypass graft; cMyBP-C, cardiac myosin-binding protein C; COPD, chronic obstructive pulmonary disease; ED, emergency department; ESM1, Endothelial cell-specific molecule 1; FABP3, fatty acid-binding protein 3; FGF23, Fibroblast growth factor 23; GDF15, Growth differentiation factor 15; Hs-cTnT, high-sensitivity cardiac troponin T; (N)STEMI, (non)-ST-elevation myocardial infarction; NT-proBNP, N-terminal probrain natriuretic peptide; PCI, percutaneous coronary intervention; proBNP, pro-B-type natriuretic peptide; t-NtproBNP, total N-terminal pro-B-type natriuretic peptide; UAP, unstable angina. Percentages may not total 100 because of rounding; * *p* values for comparison NSTEMI group versus all other diagnoses.

**Table 2:** Area under the receiver operating characteristics curve (AUC) and the optimal ROC-derived cut-off values for each biomarker.

	N	AUC (95%CI)	*p*-Value	Rule Out [Sensitivity ≥ 99.5%] (95%CI)	NPV at Sensitivity ≥ 99.5% (95%CI)	Effectiveness * [%]	PPV at Specifitiy > 95% (95%CI)
hs-cTnT [ng/L]	212 AMI, 1553 controls	0.922 (0.905–0.939)	<0.0001	≤7.9 [99.53%] (97.4–100)	99.9 (99.9–100)	44.8	63.6 (57.8–69.1)
cMyBP-C [ng/L]	212 AMI, 1553 controls	0.917 (0.896–0.938)	<0.0001	≤2.3 [99.53%] (97.4–100)	99.3 (95.2–99.9)	26.9	64.2 (58.5–69.5)
proBNP [pg/L]	212 AMI, 1553 controls	0.663 (0.628–0.698)	<0.0001	≤9.1 [99.55%] (97.5–100)	98.1 (87.8–99.7)	2.9	19.8 (13.2–28.5)
t-NtproBNP [ng/L]	212 AMI, 1553 controls	0.641 (0.604–0.677)	<0.0001	≤86.7 [99.53%] (97.4–100)	97.8 (85.9–99.7)	2.5	16.0 (9.7–25.4)
Ang2 [ng/mL]	212 AMI, 1553 controls	0.530 (0.491–0.569)	0.1379	≤0.64 [99.53%] (97.4–100)	97.1 (81.9–99.6)	2.0	87.8 (87.6–88.1)
BMP10 [ng/mL]	212 AMI, 1553 controls	0.516 (0.475–0.556)	0.4509	≤0.98 [99.53%] (97.4–100)	85.7 (42.1–98.0)	0.3	87.8 (87.6–88.0)
ESM1 [ng/mL]	212 AMI, 1553 controls	0.617 (0.576–0.658)	<0.0001	≤709.6 [99.53%] (97.4–100)	80.0 (31.0–97.3)	0.3	88.6 (88.1–89.1)
FABP3 [ng/mL]	212 AMI, 1553 controls	0.759 (0.723–0.795)	<0.0001	≤12.79 [99.52%] (97.4–100)	97.6 (85.0–99.7)	2.5	90.6 (89.9–91.3)
FGF23 [ng/mL]	212 AMI, 1553 controls	0.545 (0.504–0.586)	0.0299	≤47.08 [99.61%] (99.2–99.9)	88.0 (87.9–88.2)	6.7	91.2 (84.7–95.1)
GDF15 [pg/mL]	212 AMI, 1553 controls	0.6 (0.561–0.639)	<0.0001	≤206.6 [99.53%] (97.4–100)	75.0 (23.9–96.6)	13.3	88.4 (87.9–88.8)
Copeptin [pmoL/L]	212 AMI, 1553 controls	0.624 (0.586–0.663)	<0.0001	≤1.6 [99.53%] (97.4–100)	96.3 (78.0–99.5)	70.2	88.5 (88.0–88.9)

Abbreviations: Ang2, Angiotensin II; AUC, area under the curve; BMP10, Bone morphogenetic protein 10; cMyBP-C, cardiac myosin-binding protein C; ESM1, Endothelial cell-specific molecule 1; FABP3, fatty acid-binding protein 3; FGF23, Fibroblast growth factor 23; GDF15, Growth differentiation factor 15; Hs-cTnT, high-sensitivity cardiac troponin T; proBNP, pro-B-type natriuretic peptide; t-NtproBNP, total N-terminal pro-B-type natriuretic peptide; 95% CI, 95% confidence interval. * effectiveness indicates the proportion of patients within the cohort for whom the rule-out cut-off is applicable.

**Table 3:** Adjusted area under receiver operating characteristics curve (AUC) for biomarkers used in NSTEMI diagnosis.

Biomarker *	AUC (95%CI)	*p*-Value
hs-cTnT	0.898 (0.875–0.921)	<0.0001
cMyBP-C	0.874 (0.847–0.900)	<0.0001
proBNP	0.708 (0.674–0.742)	0.3621
t-NtproBNP	0.709 (0.675–0.743)	0.9230
Ang2	0.720 (0.685–0.754)	0.1453
BMP10	0.715 (0.680–0.749)	0.3040
ESM1	0.725 (0.693–0.757)	0.0140
FABP3	0.760 (0.728–0.793)	<0.0001
FGF23	0.726 (0.693–0.759)	0.0003
GDF15	0.714 (0.680–0.748)	0.2485
Copeptin	0.709 (0.675–0.743)	0.9628

Abbreviations: Ang2, Angiotensin II; AUC, area under the curve; BMP10, Bone morphogenetic protein 10; cMyBP-C, cardiac myosin-binding protein C; ESM1, Endothelial cell-specific molecule 1; FABP3, fatty acid-binding protein 3; FGF23, Fibroblast growth factor 23; GDF15, Growth differentiation factor 15; Hs-cTnT, high-sensitivity cardiac troponin T; proBNP, pro-B-type natriuretic peptide; t-NtproBNP, total N-terminal pro-B-type natriuretic peptide; 95% CI, 95% confidence interval. * adjusted for age in years, sex, history of myocardial infarction, diabetes, hypertension, hypercholesterolemia, current or past smoking status, GRACE score.

**Table 4:** Diagnostic performance of single and serial hs-cTnT measurement strategies for NSTEMI diagnosis.

	NPV [%] (95%CI)	Sensitivity [%] (95%CI)	PPV [%] (95%CI)	Specificity [%] (95%CI)	Effectiveness [%]
Single measurement strategy at 0-h					
hs-cTnT ≤ 14 ng/L (99th perc.)	98.7 (97.9–99.2)	92.9 (88.6–96.0)	31.5 (29.6–33.5)	72.4 (70.2–74.7)	64.6
Serial measurement strategies					
ESC 0/1 h-algorithm	100 (99.0–100)	100 (97.7–100)	30.3 (28.6–32.0)	63.5 (60.5–66.5)	54.8
ESC 0/3 h-algorithm	99.3 (98.5–99.7)	96.8 (93.1–98.8)	40.5 (37.9–43.1)	76.4 (73.8–78.8)	66

Abbreviations: CI, confidence interval; ESC, European Society of Cardiology; hs-cTnT, high-sensitivity cardiac troponin T; NPV, negative predictive value; PPV, positive predictive value.

**Table 5:** Prognostic performance of each rule-out strategy.

	Death/MI/Stroke 30 Days		Death/MI/Stroke 1 Year	
	[%] (95% CI)	[No.]	[%] (95% CI)	[No.]
Single measurement strategy at 0 h				
hs-cTnT ≤ 14 ng/L (99th perc.)	0.4 (0.1–0.9)	4/1140	2.5 (1.6–3.6)	28/1140
Copeptin < 10 pmol/L	0.6 (0.2–1.2)	7/1239	3.4 (2.4–4.6)	42/1239
MyBPC3 < 2.3 ng/L	0.2 (0.0–1.2)	1/475	1.5 (0.6–3.0)	7/475
proBNP ≤ 9.1 pg/L	0.0 (0.0–3.4)	0/52	0.0 (0.0–3.4)	0/52
t-NtproBNP ≤ 86.7 ng/L	0.0 (0.0–8.2)	0/45	2.2 (0.1–12.4)	1/45
Ang2 ≤ 0.64 ng/mL	2.9 (0.1–15.9)	1/35	2.9 (0.1–15.9)	1/35
BMP10 ≤ 0.98 ng/mL	0.0 (0.0–61.5)	0/6	0.0 (0.0–61.5)	0/6
ESM1 ≤ 709.6 ng/mL	0.0 (0.0–73.8)	0/5	0.0 (0.0–73.8)	0/5
FABP3 ≤ 12.79 ng/mL	0.0 (0.0–8.3)	0/44	0.0 (0.0–8.3)	0/44
FGF23 ≤ 47.08 ng/mL	0.0 (0.0–52.7)	0/7	28.6 (3.5–99.9)	2/7
GDF15 ≤ 206.6 pg/mL	0.0 (0.0–99.9)	0/3	0.0 (0.0–99.9)	0/3
Serial measurement strategies				
ESC 0/1 h-algorithm	0.2 (0.0–0.9)	1/646	0.8 (0.3–1.8)	5/646
ESC 0/3 h-algorithm	0.5 (0.1–1.2)	4/863	2.4 (1.5–3.7)	21/863

Abbreviations: Ang2, Angiotensin II; AUC, area under the curve; BMP10, Bone morphogenetic protein 10; cMyBP-C, cardiac myosin-binding protein C; ESC, European Society of Cardiology; ESM1, Endothelial cell-specific molecule 1; FABP3, fatty acid-binding protein 3; FGF23, Fibroblast growth factor 23; GDF15, Growth differentiation factor 15; Hs-cTnT, high-sensitivity cardiac troponin T; MI, myocardial infarction; proBNP, pro-B-type natriuretic peptide; t-NtproBNP, total N-terminal pro-B-type natriuretic peptide; 95% CI, 95% confidence interval.

## Data Availability

The data presented in this study are available on request from the corresponding author.

## Supplementary Materials

The following supporting information can be downloaded at: https://www.mdpi.com/article/10.3390/jcm14092957/s1, Table S1: Biomarker concentrations at index visit; Table S2: Cox Regression Sensitivity Analysis for 1-Year Prognostic Outcome in Patients with Confirmed NSTEMI (n = 212)—Univariable Models Using Log-Transformed Biomarker Concentrations.
