# Supplementary material for: Diagnostic and Prognostic Evaluation of Novel Biomarkers Compared to ESC 0/1 h and 0/3 h Algorithms in Patients with Suspected Non-ST-Elevation Myocardial Infarction
Source: J Clin Med. 2025 Apr 24;14(9):2957. doi: 10.3390/jcm14092957 (PMC12072832; doi:10.3390/jcm14092957)
Supplement: Supplementary file 1 [file jcm-14-02957-s001.zip › jcm-3569307-supplementary.pdf]

Diagnostic and Prognostic Evaluation of Novel Biomarkers Compared to ESC 0/1-hour and 0/3-hour Algorithms in Patients with Suspected Non-ST-Elevation Myocardial Infarction

Mustafa Yildirim [1,*], Christian Salbach [1], Matthias Mueller-Hennessen [1], Norbert Frey [1,2] and Evangelos Giannitsis [1]

[1] Department of Internal Medicine III, Cardiology, University Hospital of Heidelberg, 69120 Heidelberg, Germany; christian.salbach@med.uni-heidelberg.de (C.S.); matthias.mueller-hennessen@med.uni-heidelberg.de (M.M.-H.); norbert.frey@med.uni-heidelberg.de (N.F.); m.yildirim.2108@hotmail.com (E.G.)

[2] DZHK (German Centre for Cardiovascular Research), Standort Heidelberg/Mannheim, 69120 Heidelberg, Germany

* Correspondence: mustafa.yildirim@med.uni-heidelberg.de

Supplementary Materials

Table S1. Biomarker concentrations at index visit.

	N (measures)	Mean	Standard deviation	Median	Q1	Q3	Minimum	Maximum
hs-cTnT [ng/L]	1765	44.6 (32.6-56.7)	257.8	10 (9-10)	6 (5-6)	20 (18-22)	2.9	7067
cMyBP-C [ng/L]	1765	200.4 (144.7-256.1)	1193.8	13.6 (12.5-14-8)	5.7 (5.4-6.1)	43.4 (38.1-48.2)	0.015	29776.7
proBNP [pg/L]	1765	1019.4 (877.8-1161.1)	3035.3	189.6 (168.4-216.8)	59.5 (54.0-64.2)	903.8 (771.1-996.0)	0.321	50988.3
t-NtproBNP [ng/L]	1765	2927.5 (2574.3-3280.8)	7571.9	961.8 (881.6-1041.1)	349.1 (330.7-372.9)	2837.6 (2619.5-3162.5)	17.5	158583.9
Ang2 [ng/mL]	1765	2.4 (2.2-2.5)	2.5	1.6 (1.5-1.7)	1.15 (1.12-1.18	2.4 (2.3-2.5)	0.2	31.4
BMP10 [ng/mL]	1765	1.93 (1.89-1.97)	0.8	1.77 (1.74-1.80)	1.48 (1.47-1.51)	2.17 (2.13-2.22)	0.7	12.4
ESM1 [ng/mL]	1765	2474.2 (2397.0-2551.3)	1653.7	1893.9 (1841.1-1937.7)	1451.8 (1423.8-1483.7)	2907.9 (2740.3-3046.3)	489.1	13702.8
FABP3 [ng/mL]	1765	49.1 (34.6-63.6)	311.3	27.3 (26.6-28.0)	21.1 (20.6-21.5)	37.3 (36.4-38.3)	4.8	11403
FGF23 [ng/mL]	1765	340.3 (281.7-398.8)	1255.7	125.6 (122.1-130.0)	96.9 (94.8-99.2)	194.7 (186.4-205.4)	10	25586.3
GDF15 [pg/mL]	1765	1840.8 (1654.4.2-2027.2)	3996.0	1100.8 (1058.8-1143.4)	693.7 (668.1-726.9)	1816.8 (1693.3-1918.5)	169.7	103536.7
Copeptin [pmoL/L]	1761	13.4 (12.2-14.6)	26.0	6 (5.7-6.4)	3.6 (3.5-3.8)	11.7 (11.1-12.6)	0.7	339

Abbreviations: Ang2, Angiotensin II; BMP10, Bone morphogenetic protein 10; cMyBP-C, cardiac myosin-binding protein C; ESM1, Endothelial cell-specific molecule 1; FABP3, Fatty acid-binding protein 3; FGF23, Fibroblast growth factor 23; GDF15, Growth differentiation factor 15; Hs-cTnT, high-sensitivity cardiac troponin T; proBNP, pro-B-type natriuretic peptide; t-NtproBNP, total N-terminal pro-B-type natriuretic peptide.

Table S2. Cox Regression Sensitivity Analysis for 1-Year Prognostic Outcome in Patients with Confirmed NSTEMI (n = 212) – Univariable Models Using Log-Transformed Biomarker Concentrations.

Biomarker	HR (95% CI)	*p*-Value
hs-cTnT [ng/L]	1.91 (1.5-2.43)	<0.0001
cMyBP-C [ng/L]	1.86 (1.46-2.37)	<0.0001
proBNP [pg/L]	1.34 (1-1.79)	0.0470
t-NtproBNP [ng/L]	1.41 (1-2.01)	0.0530
Ang2 [ng/mL]	1.74 (0.72-4.21)	0.2184
BMP10 [ng/mL]	4.16 (0.37-46.56)	0.2473
ESM1 [ng/mL]	1.31 (0.62-2.76)	0.4764
FABP3 [ng/mL]	1.65 (1.27-2.14)	0.0002
FGF23 [ng/mL]	1.41 (0.9-2.2)	0.1350
GDF15 [pg/mL]	3.03 (1.82-5.06)	0.0004
Copeptin [pmoL/L]	1.68 (1.15-2.44)	0.0069

Abbreviations: Ang2, Angiotensin II; BMP10, Bone morphogenetic protein 10; cMyBP-C, cardiac myosin-binding protein C; ESM1, Endothelial cell-specific molecule 1; FABP3, Fatty acid-binding protein 3; FGF23, Fibroblast growth factor 23; GDF15, Growth differentiation factor 15; Hs-cTnT, high-sensitivity cardiac troponin T; proBNP, pro-B-type natriuretic peptide; t-NtproBNP, total N-terminal pro-B-type natriuretic peptide.
